# Is There a Difference in Factors Affecting Rest Pain and Pain Intensity during Movement at 1 Year Post–total Knee Arthroplasty?

**DOI:** 10.1298/ptr.25-E10373

**Published:** 2026-01-22

**Authors:** Yuta TOMOOKA, So TANAKA, Akira MIBU, Hirofumi YAMASHITA, Masahiro MANFUKU, Masami TOKUNAGA, Takaaki YOSHIMOTO, Tomohiko NISHIGAMI

**Affiliations:** 1) Graduate School of Comprehensive Scientific Research, Prefectural University of Hiroshima, Japan; 2) Department of Rehabilitation, Fukuoka Orthopaedic Hospital, Japan; 3) Department of Clinical Research Center, Fukuoka Orthopaedic Hospital, Japan; 4) Department of Physical Therapy, Faculty of Nursing and Rehabilitation, Konan Women’s University, Japan; 5) Department of Rehabilitation, SKY Clinic, Japan; 6) Department of Rehabilitation, Breast Care Sensyu Clinic, Japan; 7) Department of Orthopedic, Fukuoka Orthopaedic Hospital, Japan; 8) Department of Physical Therapy, Faculty of Health and Welfare, Prefectural University of Hiroshima, Japan

**Keywords:** Total knee arthroplasty, Pain intensity during movement, Rest pain, Prospective cohort study, Knee osteoarthritis

## Abstract

**Objectives:**

Total knee arthroplasty (TKA) effectively improves motor function and reduces pain in patients with osteoarthritis (OA). However, recent research emphasizes the distinction between rest pain and post-TKA pain due to their impact on treatment and recovery. This study aimed to identify factors associated with rest pain and pain intensity during movement 1 year post-TKA.

**Methods:**

A total of 126 individuals (103 females, average age: 72.3 ± 8.1 years) undergoing TKA were included. All variables were assessed 1 year postoperatively. Multiple regression analyses were performed using rest pain and pain intensity during movement as dependent variables. Independent variables included age, sex, body mass index (BMI), short form of Central Sensitization Inventory (CSI-9) score, the Fremantle Knee Awareness Questionnaire (FreKAQ) score, inappropriate femorotibial angle, and *β* angle.

**Results:**

Multiple regression analyses demonstrated that FreKAQ score was a significant independent predictor for both rest pain (*β* = 0.583, 95% confidence interval [CI]: 0.72–1.34) and pain intensity during movement (*β* = 0.486, 95% CI: 0.72–1.52). Additionally, CSI-9 score (*β* = 0.183, 95% CI: 0.02–0.93) and the *β* angle (*β* = −0.218, 95% CI −3.17 to −0.60) were significant independent predictors for pain intensity during movement only.

**Conclusions:**

Specific treatments addressing disturbed body perception may benefit patients with rest pain. Conversely, pain intensity during movement was found to be influenced by various factors, including coronal alignment of the knee joint, central sensitization (CS)-related symptoms, and disturbed body perception. This suggests a need for more comprehensive treatment strategies for pain intensity during movement.

## Introduction

Total knee arthroplasty (TKA) is one of the most commonly utilized orthopedic surgeries for the improvement of motor function and pain relief in knee osteoarthritis (OA)^1)^. Despite its success in removing the damaged surfaces of the knee joint, approximately 20% of patients experience persistent postoperative pain, significantly impacting their daily functioning and quality of life^2–5)^. Therefore, identifying factors contributing to this complication is crucial.

Several investigations have examined the correlation between persistent pain and various factors measured at 1 year post-TKA, including radiographic abnormalities (inappropriate femorotibial angle [FTA] and medial coronal inclination of the tibial component), psychological factors (anxiety, depression, and pain catastrophizing), and central sensitization (CS)-related symptoms^6–8)^. Recent reviews have emphasized the importance of distinguishing between rest pain and pain intensity during movement due to their distinct underlying mechanisms and clinical implications^9,10)^. Pain intensity during movement occurs during and immediately after active or passive movement^10)^ and is often more severe than rest pain^9,10)^. Previous studies suggest that pre-TKA pain sensitivity predicts pain intensity during movement but not rest pain^11)^. Additionally, transcutaneous electrical stimulation effectively reduces pain intensity during walking and improves gait speed, but does not alleviate rest pain^12)^. The study further indicated that opioids, commonly used for severe postoperative pain, are effective for rest pain but may have limited effectiveness for early postoperative pain intensity during movement^13)^. These findings suggest the need for tailored treatment approaches for each pain type.

Interestingly, a previous study reported that pain intensity during movement demonstrated a stronger association with chronic pain post-TKA than rest pain^14)^. However, to the best of our knowledge, no cross-sectional studies have examined the factors influencing rest pain and pain intensity during movement at 1 year postoperatively. Cross-sectional studies provide valuable insights into the relationships between various factors and diseases at specific time points. Therefore, such studies can help determine the factors associated with rest pain and pain intensity during movement, which are pertinent for developing new treatment strategies.

The present study aimed to identify factors related to rest pain and pain intensity during movement 1 year post-TKA, as well as to investigate the characteristics of groups classified based on pain type. We hypothesized that different factors influence each pain type, with more factors associated with pain intensity during movement.

## Methods

### Study design and participants

This was a cross-sectional study including patients with knee OA who underwent TKA at a hospital between October 2019 and September 2021. Inclusion criteria were age <90 years and primary TKA for OA. Patients with rheumatoid arthritis, lateral knee OA, previous TKA on the contralateral knee, who were unable to complete the questionnaire, a history of fracture around the knee joint, and who were followed up at a different hospital were excluded.

### Procedure

All patients underwent surgery at the same institution, which was performed by 13 surgeons, following a standardized protocol. Tourniquets were used for bleeding control and implant cementation during surgery. General anesthesia was used for all patients, and depending on individual needs, epidural, spinal, or intravenous block anesthesia was added. Primary TKA was performed using posterior-stabilized (PS) components (Persona; Zimmer Biomet, Warsaw, IN, USA) or bi-cruciate-stabilized (BCS) components (or Journey Ⅱ; Smith & Nephew, London, UK). The physicians selected the component types as follows: Persona is designed to better align with the patient’s anatomical structure, while Journey Ⅱ is intended to achieve more physiological kinematics. Skin incisions were an anterior straight midline incision or a medial curved incision, and surgical approaches were a medial parapatellar approach or a midvastus approach. Patellar replacement was performed on all patients. Before closure, all patients received intra-articular cocktail injections (20 mL saline, 20 mL 0.75% ropivacaine, 1 mL 4 mg dexamethasone, and 0.1 mL morphine).

A postoperative multimodal pain control protocol was initiated on the same day as surgery. This protocol included continuous epidural anesthesia or intravenous fentanyl patient-controlled analgesia until the following day. Scheduled systemic analgesia included acetaminophen and non-steroidal anti-inflammatory drugs (NSAIDs), administered as needed. Specifically, acetaminophen was administered according to standard pharmacological guidelines for adults, which generally permit 300–1000 mg of acetaminophen per administration, with a dosing interval of 4–6 h or longer, up to a maximum total daily dose of 4000 mg. The NSAID administered was loxoprofen, 60 mg, up to three times daily as needed. For instances of severe pain, a 50 mg dose of the weak opioid tramadol was added judiciously. Medications were adjusted based on patient reports and clinical judgment; however, the study protocol did not include a systematic, objective assessment of specific pain intensity during this early postoperative phase.

Early range-of-motion (ROM) exercises and weight-bearing with a walker were initiated the day after surgery. All patients underwent a postoperative physical therapy program, which followed guidelines aimed at improving ROM and strength, reducing pain, and normalizing gait^15)^. Each exercise was repeated 10 times, with intensity and volume adjusted based on clinical condition. Physical therapy sessions were conducted for 30 minutes daily for 5 weeks by an experienced physiotherapist (>15 years of experience). At the end of the 5-week period, the patient was discharged from the hospital. None of the patients received outpatient physical therapy after discharge.

### Measurements

Demographic data (age and sex), body mass index (BMI), pain catastrophizing, knee-specific body perception, CS-related symptoms, pain intensity, radiographic data, knee joint extension ROM, knee joint flexion ROM, 10-Meter Walk test (10MWT), and knee-specific disability were collected at 1 year postoperatively. All questionnaires were handed to the patients by an assistant different from the interventionists involved in their care. Patients completed these questionnaires independently. All evaluations took approximately 30 minutes.

#### Pain intensity

At the 1-year postoperative outpatient visit, rest pain and pain intensity during movement were assessed using a single 0–100-mm Visual Analog Scale (VAS). Participants were instructed to recall typical daily activities (e.g., level walking, sit-to-stand, stair negotiation) rather than perform a standardized provocation task with immediate on-task ratings. Rest pain was evaluated by asking participants, “What is the intensity of your knee pain with rest?” Pain intensity during movement was similarly evaluated with the following question: “What is the intensity of your knee pain with movement?”^16)^.

#### Pain catastrophizing

The Pain Catastrophizing Scale (PCS) is designed to assess an individual’s thoughts and feelings when experiencing pain. This 13-item scale evaluates magnification, rumination, and helplessness regarding pain, with higher scores indicating higher levels of pain catastrophizing^17)^. The Japanese version of the PCS, which is used in the present study, has demonstrated internal consistency and criterion validity^18,19)^.

#### Knee-specific body perception

The Fremantle Knee Awareness Questionnaire (FreKAQ)^20)^, adapted from the Fremantle-Back Awareness Questionnaire (FreBAQ)^21)^, measures self-reported knee-specific body perception in individuals with knee pain. This questionnaire comprises 9 items that were rated on a five-point Likert scale, ranging from 0 (never) to 4 (always). Higher FreKAQ scores indicate a more disturbed body perception, including neglect-like symptoms, reduced proprioceptive acuity, and altered perceptions of body part shape and size. The Japanese version of this scale, which is used in the present study, has also been validated^20)^.

#### CS-related symptoms

The short form of Central Sensitization Inventory (CSI-9) is a clinically refined version of the original CSI^22)^, serving as a screening tool to identify individuals with CS-related symptoms. The CSI-9 comprises 9 items rated on a five-point Likert scale, ranging from 0 (never) to 4 (always) for a maximum total score of 36. Higher scores indicate a greater degree of self-reported CS-related symptoms. The Japanese version of this scale has similarly been translated and validated for use.

#### Radiographic data

The severity of knee OA prior to TKA was evaluated using the Kellgren–Lawrence classification. Postoperative lower limb alignment was assessed by measuring the FTA, defined as the angle between the anatomical axes of the femur and tibia on standing radiographs. FTA was measured using full-length, weight-bearing lower limb radiographs. An inappropriate FTA was defined as an FTA >178° or <170°^23)^. The implant position angle was measured using the Knee Society’s roentgenographic evaluation system^24)^, focusing on the coronal medial inclination of the tibial component (*β*), an angle previously identified as indicative of abnormal positioning^6)^. The *β* angle is measured between the anatomical axis of the tibia and the line parallel to the tibial tray^6)^. The *β* angle was measured on standardized anteroposterior radiographs by 1 physical therapist and 1 orthopedic surgeon. The inter-rater agreement between the 2 examiners had been verified in a preliminary internal assessment using similar cases at our institution, showing excellent reliability (intraclass correlation coefficient [ICC] of 0.93 for ICC [1,2], unpublished data). Importantly, this reliability study was conducted on a cohort of 30 patients who had undergone TKA, separate from the present sample, to avoid circularity. The full reliability dataset is provided in Supplementary Table 1.

#### Knee-specific disability

The Oxford Knee Score (OKS) was utilized to assess knee-specific functional impairment, having been validated for its reliability, validity, and sensitivity to change in post-arthroplasty patients^25)^. The OKS provides a concise, patient-reported evaluation of knee function through 12 items rated on a Likert scale, ranging from 0 (total disability) to 4 (no disability). Its summed score (0–48) reliably reflects the degree of disability, with higher values corresponding to superior functional status.

### Sample size

A sample size of 109 individuals for multiple regression analyses was calculated using the G*Power ver.3.1 (Heinrich-Heine-Universität Düsseldorf, Düsseldorf, Germany) with the following parameters: α error = 0.05, power = 0.8, effect size = 0.15, and number of predictors = 8. We chose *f*^2^ = 0.15 as a pragmatic and conservative assumption recommended in practical guidance on sample-size planning^26)^, and because prior literature in this field often reports small-to-moderate associations between pain-related psychometric constructs and disability.

### Statistical analyses

At the 1-year follow-up, patients were categorized into two groups based on their pain intensity during movement VAS scores—those with scores ≥30 mm were included in the chronic postsurgical pain (CPSP) group, and those with scores <30 mm were included in the non-CPSP group. In addition, comparisons were made between the two implant types (Persona vs. Journey II). To examine potential sex-related differences, we compared males and females. Nominal variables were analyzed using the chi-squared test, while continuous variables were first assessed for normality (via histogram inspection) and then analyzed using independent t-tests for normally distributed data or Mann–Whitney U tests for non-normally distributed data.

Continuous variables were presented as means ± standard deviation, while categorical variables were presented as frequencies (N) and percentages (%). Correlation coefficients between continuous variables and pain were analyzed using Pearson’s correlation analysis. To mitigate inflation of type I error due to multiple testing, we controlled the familywise error rate (FWER) using the Bonferroni correction across m = 8 correlation tests. The significance threshold was set at α_adj = 0.05/8 = 0.006 (two-sided), thereby reducing the risk of spurious findings arising by chance.

To investigate the association between rest pain, pain intensity during movement, and potential influencing factors, multiple linear regression analyses were conducted. Rest pain and pain intensity during movement served as dependent variables, whereas age, sex, BMI, CSI-9, FreKAQ, FTA, and *β* angle, all assessed at 1 year, served as independent variables. All statistical analyses were performed using SPSS Statistics ver.26 (IBM, Armonk, NY, USA), and statistical significance was set at *p* <0.05. The variance inflation factor (VIF) was determined to investigate multicollinearity, which was considered present if VIF >5.

## Results

### Demographics and clinical characteristics of individuals at 1 year post-TKA

**Fig. 1:**
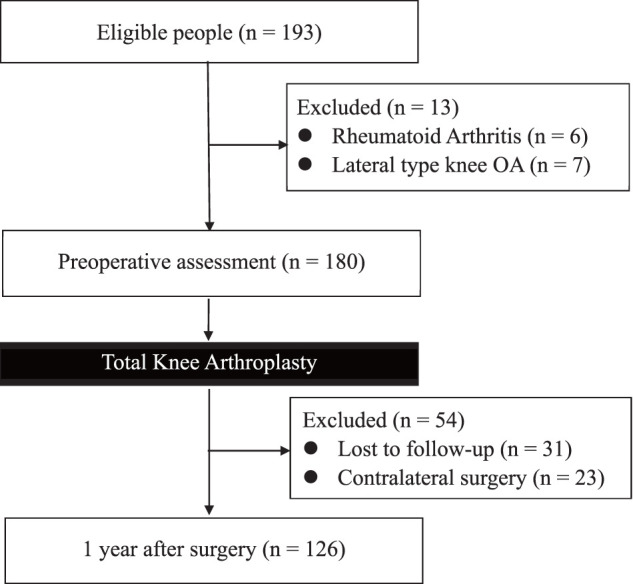
Study flow diagram for participant inclusion and analysis at 1 year post-TKA. Of the 193 patients who consented to participate, 126 completed the 1-year assessment and were included in the final analyses. Patients who were lost to follow-up, were followed at a different hospital, had undergone prior contralateral TKA, or otherwise did not meet the eligibility criteria were excluded. TKA, total knee arthroplasty; OA, osteoarthritis

A total of 193 individuals consented to participate in the study prior to TKA. Patients with rheumatoid arthritis (n = 6), lateral knee OA (n = 7), prior TKA on the contralateral knee (n = 23), and those who followed up at another hospital or had no follow-up before 1-year assessment (n = 31) were all excluded. Thus, only 126 patients completed the final assessment at 1 year postoperatively (Fig. 1). Table 1 summarizes the demographics of the included participants at 1 year post-TKA (Table 1). Comparison between the CPSP (pain intensity during movement VAS ≥30 mm) and the non-CPSP group revealed significant differences were observed in pain intensity during movement, rest pain, FreKAQ scores, CSI-9 scores, and OKS scores (Table 2). Between-group differences are reported as effect sizes—Hedges’ g (normal), Cliff’s δ (non-normal), and odds ratios (binary)—all with 95% confidence intervals (CIs), noting the small CPSP subgroup (n = 12). Furthermore, when comparing patients by implant type (Persona vs. Journey II), no significant differences were found in either preoperative or 1-year postoperative variables (Table 3). In the sex subgroup comparison, only 10MWT differed significantly between males and females, whereas pain intensity outcomes showed no significant sex differences. Detailed statistics are provided in Supplementary Table 2.

**Table 1:** Demographic and clinical characteristics of patients at 1 year post-TKA (n = 126)

Characteristics	
Age (years) (SD)	71.7 (8.1)
Sex (women) (%)	103 (81.7%)
BMI (kg/m^2^) (SD)	26.7 (4.1)
TKA type (%)	
Persona	67 (55.2%)
Journey Ⅱ	59 (44.8%)
Skin incision	
Anterior straight midline incision	106 (84.1%)
Medial curved incision	20 (15.9%)
Surgical approaches	
Medial parapatellar approach	115 (90.9%)
Midvastus approach	11 (9.1%)
Pain intensity (VAS: 0–100)	
Rest pain, median (range)	2 (0– 60)
Pain intensity during movement, median (range)	6 (0– 60)
PCS (0–52) (SD)	6.4 (8.1)
FreKAQ (0–36) (SD)	5.6 (5.6)
CSI-9 (0–36) (SD)	5.0 (6.2)
Inappropriate FTA (%)	29 (23.0%)
*β* angle (°) (SD)	88.9 (1.5)
Inappropriate *β* angle (%)	12 (9.5%)
Knee extension ROM (°) (SD)	−3.8 (4.8)
Knee flexion ROM (°) (SD)	123.3 (9.9)
10MWT (s)	6.9 (1.5)
OKS (0–48) (SD)	40.6 (5.8)

Data are mean ± SD or N (%), except for pain intensity values, which are reported as median [min–max] due to their non-normal distribution.

Inappropriate FTA was defined as femorotibial angle >178° or <170°. Inappropriate *β* angle is defined as 90° ± 3°.

TKA, total knee arthroplasty; SD, standard deviation; BMI, body mass index; VAS, Visual Analog Scale; PCS, pain catastrophizing scale; FreKAQ, Fremantle Knee Awareness Questionnaire; CSI-9, the short form of Central Sensitization Inventory; FTA, femorotibial angle; ROM, range of motion; 10MWT, 10-Meter Walk Test; OKS, Oxford Knee Score

**Table 2:** Comparison of non-CPSP and CPSP groups

Characteristics	Non-CPSP group (n = 114)	CPSP group (n = 12)	Effect size	95% CI	*p*-Value
Age (years) (SD)	72.4 (7.9)	71.3 (9.5)	0.13	[−3.78, 5.95]	0.66
Sex (women) (%)	93 (81.6%)	10 (83.3)	1.13	[0.23, 5.54]	0.62
BMI (kg/m^2^) (SD)	26.8 (4.2)	26.2 (3.1)	0.16	[−1.84, 3.14]	0.61
Pain intensity (VAS: 0–100)					
Rest pain (IQR)	2.0 (0, 6.0)	23.0 (1.5, 27.0)	−0.46	[−24, −1]	0.008*
Pain intensity during movement (IQR)	4.5 (1.0 ,11.0)	40.5 (36.5, 50.0)	–	[−40, −30]	<0.001*
PCS (0–52) (IQR)	3.0 (0, 10.0)	12.0 (4.0, 14.5)	−0.34	[−10, 0]	0.05
FreKAQ (0–36) (IQR)	3.5 (1.0, 9.0)	11.0 (5.0, 15.5)	−0.57	[−11, −3]	0.001*
CSI-9 (0–36) (IQR)	5.0 (2.0, 8.0)	9.5 (5.5, 14.0)	−0.46	[−7, −1]	0.009*
Inappropriate FTA (%)	27 (23.7%)	2 (16.7%)	0.83	[0.21−3.21]	0.56
*β* angle (°) (SD)	89.0 (1.5)	87.9 (1.1)	0.77	[−0.24, 2.00]	0.13
Knee extension ROM (°) (SD)	−3.7 (4.8)	−4.5 (4.9)	0.09	[−2.47, 3.28]	0.86
Knee flexion ROM (°) (SD)	123.7 (9.8)	119.5 (11.7)	0.15	[−2.99, 8.84]	0.98
10MWT (s) (SD)	6.9 (1.5)	7.5 (1.8)	−0.24	[−1.32, 0.58]	0.68
OKS (0–48) (SD)	41.4 (4.9)	32.4 (8.0)	1.73	[5.87, 12.13]	0.003*

Data in the table are presented using N (%) for nominal variables, and for continuous variables, the mean ± SD is used when the data follow a normal distribution, while the median and IQR are reported when the distribution is non-normal.

Inappropriate FTA was defined as femorotibial angle >178° or <170°.

*p <0.05.

Because CPSP was defined using movement-evoked pain (movement VAS [MVAS] ≥30 mm), the MVAS row is tautological, and its effect size approaches −1; it is reported for completeness and should not be interpreted.

CPSP, chronic postsurgical pain; CI, confidence interval; SD, standard deviation; BMI, body mass index; VAS, Visual Analog Scale; IQR, interquartile range; PCS, pain catastrophizing scale; FreKAQ, Fremantle Knee Awareness Questionnaire; CSI-9, the short form of Central Sensitization Inventory; FTA, femorotibial angle; ROM, range of motion; 10MWT, 10-Meter Walk Test; OKS, Oxford Knee Score

**Table 3:** Comparison of preoperative and postoperative variables by implant type (Persona vs. Journey Ⅱ)

Characteristics	Persona (n = 67)	Journey Ⅱ (n = 59)	*p*-Value
Age (years) (SD)	73.0 (8.8)	71.5 (7.1)	0.32
Sex (women) (%)	52 (77.6%)	51 (86.4)	0.06
BMI (kg/m^2^) (IQR)	26.3 (24.4, 28.2)	26.1 (24.3, 28.4)	0.78
Pain intensity (VAS: 0–100)			
Rest pain (IQR)	2.0 (0, 5.0)	2.0 (0, 9.0)	0.40
Pain intensity during movement (IQR)	4.0 (1.0, 14.0)	6.0 (1.0, 13.0)	0.53
PCS (0–52) (IQR)	2.0 (0, 9.0)	4.0 (0, 11.0)	0.13
FreKAQ (0–36) (IQR)	4.0 (1.0, 9.0)	4.0 (1.0, 9.0)	0.78
CSI-9 (0–36) (IQR)	5.0 (3.0, 8.0)	5.0 (2.0, 9.0)	0.92
Inappropriate FTA (%)	16 (25.3%)	12 (20.3%)	0.39
*β* angle (°) (SD)	88.8 (1.5)	89.0 (1.5)	0.43
Knee extension ROM (°) (SD)	−4.8 (5.1)	−2.7 (4.2)	0.49
Knee flexion ROM (°) (SD)	121.3 (9.7)	125.7 (9.8)	0.90
10MWT (s) (SD)	6.9 (1.6)	6.9 (1.4)	0.54
OKS (0–48) (SD)	41.3 (5.1)	39.7 (6.6)	0.28

Data in the table are presented using N (%) for nominal variables, and for continuous variables, the mean ± SD is used when the data follow a normal distribution, while the median and IQR is reported when the distribution is non-normal.

SD, standard deviation; BMI, body mass index; IQR, interquartile range; VAS, Visual Analog Scale; PCS, pain catastrophizing scale; FreKAQ, Fremantle Knee Awareness Questionnaire; CSI-9, the short form of Central Sensitization Inventory; FTA, femorotibial angle; ROM, range of motion; 10MWT, 10-Meter Walk Test; OKS, Oxford Knee Score

### Correlations and multiple regression analyses of rest pain and pain intensity during movement at 1 year post-TKA

Pearson’s correlation analysis demonstrated that rest pain was positively correlated with FreKAQ (*r* = 0.584, *p* <0.001) and CSI-9 scores (*r* = 0.304, *p* = 0.001). Meanwhile, pain intensity during movement was positively correlated with PCS (*r* = 0.265, *p* = 0.003), FreKAQ (*r* = 0.547, *p* <0.001), and CSI-9 scores (*r* =0.375, *p* <0.001) (Table 4).

**Table 4:** Correlation of rest pain and pain intensity during movement at 1 year post-TKA

	Rest pain		Pain intensity during movement	
	*r*	*p*-Value	*r*	*p*-Value
Age	0.022	0.81	−0.063	0.48
BMI	0.052	0.56	0.061	0.50
PCS	0.234	0.008	0.265	0.003*
FreKAQ	0.584	<0.001*	0.547	<0.001*
CSI-9	0.304	0.001*	0.375	<0.001*
*β* angle	−0.076	0.40	−0.189	0.034

*p <0.006.

TKA, total knee arthroplasty; BMI, body mass index; PCS, Pain Catastrophizing Scale; FreKAQ, Fremantle Knee Awareness Questionnaire; CSI-9, the short form of Central Sensitization Inventory

Both regression models with rest pain and pain intensity during movement as dependent variables were statistically significant (*F* = 8.1, *p* <0.001, *R*^2^ = 0.36, adjusted *R*^2^ = 0.31; *F* = 8.8, *p* <0.001, *R*^2^ = 0.37, adjusted *R*^2^ = 0.33, respectively). The observed overall effect sizes (*f*^2^), calculated from the adjusted *R*^2^ values, were 0.45 for the rest-pain model and 0.49 for the pain intensity during movement model. These correspond to large effect sizes and were greater than the anticipated effect size (*f*^2^ = 0.15) used in a priori power analysis. Multiple regression analyses identified the FreKAQ score as a significant and independent predictor of rest pain (*β* = 0.583, 95% CI: 0.72–1.34) and pain intensity during movement (*β* = 0.486, 95% CI: 0.72–1.52). Furthermore, the CSI-9 score (*β* = 0.183, 95% CI: 0.02–0.93) and the *β* angle (*β* = −0.218, 95% CI: −3.17 to −0.60) were significantly and independently associated with pain intensity during movement (Table 5). VIF analysis indicated no multicollinearity among the independent variables.

**Table 5:** Multiple regression analysis of rest pain and pain intensity during movement at 1 year post-TKA

Independent variables	*B*	*β*	95% CI	*p*-Value	VIF
Rest pain (*F* = 8.1, *p* <0.001, *R*^2^ = 0.36, adjusted *R*^2^ = 0.31)					
Age	−0.01	−0.010	[−0.23, 0.47]	0.90	1.19
Sex	1.17	0.046	[−2.65, 4.98]	0.55	1.04
BMI	−0.17	−0.070	[−0.56, 0.23]	0.40	1.26
PCS	−0.04	−0.034	[−0.25, 0.17]	0.70	1.37
FreKAQ	1.03	0.583	[0.72, 1.34]	<0.01*	1.45
CSI-9	0.12	0.060	[0.23, 0.47]	0.51	1.47
Inappropriate FTA	0.34	0.014	[−3.20, 3.87]	0.85	1.06
*β* angle	−0.64	−0.097	[−1.64, 0.36]	0.21	1.06
Pain intensity during movement (*F* = 8.8, *p* <0.001, *R*^2^ = 0.37, adjusted *R*^2^ = 0.33)					
Age	−0.14	−0.087	[−0.39, 0.11]	0.28	1.19
Sex	−1.21	−0.036	[−6.11, 3.69]	0.63	1.04
BMI	−0.32	−0.102	[−0.82, 0.19]	0.21	1.26
PCS	−0.01	−0.004	[−0.28, 0.27]	0.97	1.37
FreKAQ	1.12	0.486	[0.72, 1.52]	<0.01*	1.45
CSI-9	0.47	0.183	[0.02, 0.93]	0.041*	1.47
Inappropriate FTA	1.66	0.055	[−2.88, 6.21]	0.47	1.06
*β* angle	−1.89	−0.218	[−3.17, 0.60]	<0.01*	1.06

Inappropriate FTA was defined as femorotibial angle >178° or <170.

*p ≤0.05.

TKA, total knee arthroplasty; *B*, unstandardized regression coefficient; CI, confidence interval; VIF, variance inflation factor; BMI, body mass index; PCS, pain catastrophizing scale; FreKAQ, Fremantle Knee Awareness Questionnaire; CSI-9, the short form of Central Sensitization Inventory; FTA, femorotibial angle

## Discussion

This study investigated the influence of various factors, including demographic characteristics, pain-related measures, and radiological imaging, on rest pain and pain intensity during movement 1 year post-TKA. Multiple regression analysis showed that both types of pain were associated with disturbed body perception, whereas CS-related symptoms and postoperative coronal alignment of the knee joint only affected pain intensity during movement. Our findings revealed a significant difference in 1-year OKS between patients with CPSP and those without, indicating that functional impairment, as measured using the OKS, is distinctly associated with the presence of persistent pain. This underscores the intricate relationship between pain experience and functional capacity following TKA, emphasizing the necessity of integrating both pain and functional assessments for a holistic understanding of patient recovery and guiding tailored rehabilitation strategies^27)^. Self-reported body perception was associated with both rest pain and pain intensity during movement at 1 year post-TKA. Previous research reported a similar association in patients with knee OA, where disturbed body perception correlated with pain intensity, disability, and reduced relief of pain intensity^16,28)^. Moreover, Nishigami et al.^29)^ reported that cognitive factors and disturbed body perception had a greater impact on knee-related disability in knee OA compared to the severity of radiographic OA. These studies suggest a potential link between pain and disturbed body perception in patients with knee OA.

In the context of TKA, Hirakawa et al.^30)^ reported that the neglect-like symptoms questionnaire developed by Frettlöh et al.^31)^ was significantly associated with pain at 6 weeks postoperatively. Research on proprioception post-TKA remains inconclusive, with studies reporting a decline^32–34)^ or improvement in proprioception^35–39)^. Perceived knee enlargement post-TKA has also been associated with poor patient satisfaction and functional disability^40)^. Since the FreKAQ encompasses items related to neglect-like symptoms, proprioception, and perceived body shape and size, it is highly likely that disturbed body perception contributes to pain and disability in both TKA and knee OA. Notably, our study showed that distorted body perception was associated with rest pain and pain intensity during movement. Previous studies on musculoskeletal disorders have yielded inconclusive findings between disturbed body perception and both pain types. Reports utilizing a body perception questionnaire tailored to the knee, neck, and lower back demonstrated associations with pain intensity during movement but not rest pain^21,41,42)^. The reason why surgical management influences the association between body perception and resting pain remains unclear and warrants further investigation.

As hypothesized, various factors were found to influence rest pain and pain intensity during movement. In addition to the FreKAQ score, pain intensity during movement was also associated with the CSI-9 score and *β* angle. A review of pain intensity during movement suggests that both central and peripheral mechanisms play a role^43)^. A recent systematic review further supports the strong link between the CSI scores and more severe, chronic pain in patients post-TKA^44)^. However, this review did not distinguish between rest pain and pain intensity during movement. Others reported that rest pain is more affected by central mechanisms than pain intensity during movement in the early postoperative period^45)^. Our findings are consistent with recent evidence implying a stronger influence of central mechanisms on pain intensity during movement than on rest pain at 1 year post-TKA^43)^. Also, a systematic review showed an association between pain and catastrophizing, but did not include patients with TKA^46)^. Pain intensity during movement at 1 year post-TKA may not be cross-sectionally associated with catastrophizing.

While most studies on post-TKA pain neglected to focus on radiographic alignment^47–49)^, previous research has established specific criteria for acceptable tibial component positioning. In previous studies, tibial component alignment in the coronal plane was considered acceptable if it deviated no more than 3° from the neutral position (i.e., 90° ± 3°)^50)^. In the present study, the *β* angle was also assessed, suggesting that implantations with deviations beyond this range may be associated with pain intensity during movement. One study showed the impact of the *β* angle on chronic pain at 1 year postoperatively^6)^ and on painful TKA with inappropriate FTA^51)^. Our results identified the *β* angle as an independent factor for pain intensity during movement at 1 year. In our data, a 1° change in the *β* angle was associated with an approximately 1.9-mm change on the 0–100-mm VAS for pain intensity during movement. Given that an MCID of about 18 mm has been reported post-TKA^52)^, an alignment change of roughly 9–10° would be required to approach an MCID-level difference. Accordingly, although the *β* angle remained an independent predictor of pain intensity during movement, its stand-alone clinical impact within usual alignment ranges (90° ± 3°) is likely modest. Future studies should therefore consider the role of alignment in addressing pain intensity during movement post-TKA. Some patients with persistent postoperative pain may be classified as having nociplastic pain. Nociplastic pain was first defined in 2016 as “pain that arises from altered nociception despite no clear evidence of actual or threatened tissue damage causing the activation of peripheral nociceptors or evidence for disease or lesion of the somatosensory system causing the pain”^53)^. Miki et al. developed a treatment algorithm for persistent postoperative pain post-TKA and proposed that surgical or pharmacological methods, including opioid therapy for organic pain, such as intra-articular infection and periarticular fractures; for neuropathic pain, NSAIDs, pregabalin, and anticonvulsants; and for nonorganic pain, psychiatric approaches such as antidepressant therapy and cognitive behavioral therapy, should be implemented^54)^. In the present study, nonorganic interventions may be similarly effective for pain intensity during movement affected by CS syndrome (CSS) related to nociplastic pain.

### Clinical implications

Given our results on the impact of various factors on rest pain and pain intensity during movement, targeted treatment approaches for each pain type are crucial. For rest pain, specific treatments that target disturbed body perception may be beneficial. Pain intensity during movement, however, is associated with multiple factors, including coronal alignment of the knee joint, CS-related symptoms, and disturbed body perception, suggesting the need for more comprehensive treatment strategies. While component repositioning might not be feasible, pain neuroscience education can be valuable in the multimodal approach for chronic pain due to CS^55)^. A randomized clinical trial involving patients with chronic low back pain demonstrated that graded sensorimotor retraining for disturbed body perception significantly improved pain intensity after 18 weeks^56)^. The effect of graded sensorimotor retraining in TKA remains unknown, warranting further investigation on this matter.

### Limitations

Despite the valuable insights offered in this study, several limitations must be acknowledged. First, although we evaluated CS-related symptoms, we did not assess neurophysiological measurements, such as pressure pain threshold, temporal summation, and conditioned pain modulation. Thus, the specific mechanisms by which CS might influence rest pain and pain intensity during movement remain unclear. Second, as this was a single-center study including a higher proportion of females aged >80 years (21.4%), the generalizability of the findings may be limited. Third, pain intensity during movement in this study was assessed at the 1-year postoperative visit using a single 0–100 mm VAS anchored to typical daily activities, rather than a standardized provocation protocol with immediate on-task ratings. This non-standardized approach introduces between-participant variability in the eliciting activity and recall bias, leaves test–retest reliability unknown, and may attenuate associations and limit comparability with more recent studies employing fixed tasks and standardized timing. As such, further studies incorporating standardized methods to assess pain intensity during movement are warranted. Fourth, the cross-sectional study design at a single time point (1 year post-TKA) prevented the analysis of changes in variables over time, such as changes in FreKAQ scores and their association with pain outcomes. Future longitudinal studies are needed to investigate the temporal relationship between disturbed body perception and persistent pain. Fifth, the CPSP subgroup was small (n = 12), which limits statistical power and widens CIs in between-group comparisons. Accordingly, these subgroup results should be interpreted cautiously and regarded as exploratory/hypothesis-generating. We therefore emphasize effect sizes with 95% CIs rather than dichotomous *p* values, and replication in larger, more balanced samples is warranted. Sixth, the absence of between-group differences across outcomes in the Persona versus Journey II comparison suggests that implant design is unlikely to account for the observed associations. Nevertheless, unmeasured surgical factors may still contribute to residual confounding, which future studies should address. Seventh, because this study was cross-sectional and conducted 1 year post-TKA, causal inferences cannot be established. Future longitudinal cohort or interventional studies are warranted to elucidate causal relationships. Eighth, body composition parameters (lean and fat mass) and the presence of sarcopenia or malnutrition were not systematically available for all participants. Future studies should incorporate standardized assessments of body composition, nutritional status, and related covariates.

## Conclusions

This study showed that distinct factors affect rest pain and pain intensity during movement in patients with knee OA who underwent TKA 1 year postoperatively. While rest pain may benefit from targeted interventions for disturbed body perception, pain intensity during movement requires a more comprehensive approach due to the involvement of multiple factors, such as coronal alignment of the knee joint, CS-related symptoms, and disturbed body perception.

## Data Availability

Datasets generated and analyzed in this study are available from the corresponding author upon reasonable request.

## Supplementary Materials

Supplementary Table 1: Inter-rater *β*-angle measurements.

Supplementary Table 2: Comparison of sex differences.
