# Supplementary material for: Is There a Difference in Factors Affecting Rest Pain and Pain Intensity during Movement at 1 Year Post–total Knee Arthroplasty?
Source: Phys Ther Res. 2026 Jan 22;29(1):6–15. doi: 10.1298/ptr.25-E10373 (PMC13143134; doi:10.1298/ptr.25-E10373)
Supplement: Supplementary file 1 — Supplementary Table 1: Inter-rater β-angle measurements. [file ptr-29-06-s001.pdf]

Supplementary 1. Inter-rater *β*-angle measurements (independent dataset, n = 30).

Case ID	PT, *β* (°)	OS, *β* (°)	Mean *β* (°)	Abs. diff (°)
01	93.7	93.8	93.8	0.1
02	94.3	94.8	94.6	0.5
03	86.0	88.1	87.0	2.1
04	94.1	92.4	93.2	1.7
05	91.6	92.5	92.0	0.9
06	89.2	90.1	89.6	0.9
07	94.1	92.8	93.4	1.3
08	89.5	89.7	89.6	0.2
09	87.1	86.8	86.9	0.3
10	90.9	90.3	90.6	0.6
11	89.3	86.3	87.8	3.0
12	93.7	93.8	93.8	0.1
13	83.6	84.8	84.2	1.2
14	89.0	90.4	89.7	1.4
15	86.4	81.9	84.2	4.5
16	91.6	90.2	90.9	1.4
17	94.5	93.1	93.8	1.4
18	90.3	90.7	90.5	0.4
19	91.9	93.3	92.6	1.4
20	85.2	86.3	85.8	1.1
21	88.7	91.1	89.9	2.4
22	89.7	90.3	90.0	0.6
23	89.4	87.9	88.6	1.5
24	87.3	87.8	87.6	0.5
25	90.9	90.3	90.6	0.6
26	88.4	85.2	86.8	3.2
27	89.2	90.0	89.6	0.8
28	90.1	90.8	90.4	0.7
29	88.3	88.7	88.5	0.4
30	86.9	86.5	86.7	0.4

PT = physical therapist; OS = orthopedic surgeon; Mean β = arithmetic mean of PT and OS; Abs. diff = |PT − OS|. All angles in degrees (°).

Summary (Abs. diff): mean 1.2°, SD 1.0°, median (IQR) 0.9° (0.5–1.4°), range 0.1–4.5°. Inter-rater reliability: ICC (1,2) = 0.93 (unpublished); see Methods.
