# Supplementary material for: Is There a Difference in Factors Affecting Rest Pain and Pain Intensity during Movement at 1 Year Post–total Knee Arthroplasty?
Source: Phys Ther Res. 2026 Jan 22;29(1):6–15. doi: 10.1298/ptr.25-E10373 (PMC13143134; doi:10.1298/ptr.25-E10373)
Supplement: Supplementary file 2 — Supplementary Table 2: Comparison of sex differences. [file ptr-29-06-s002.pdf]

Supplementary 2. Comparison of Sex Differences

Characteristics	male (n = 23)	female (n = 103)	*p*
Age (years) (SD)	71.7 (9.2)	72.5 (7.8)	0.69
BMI (kg/m^2) (SD)	26.5 (4.1)	26.8 (4.2)	0.61
Pain intensity (VAS: 0-100)			
Rest pain: (IQR)	1.5 (0, 7.5)	2.0 (0, 7.0)	0.90
Pain intensity during movement: (IQR)	6.0 (1.5, 18.0)	6.0 (1.0, 12.8)	0.67
PCS (0-52) (IQR)	4.0 (1.0, 12.5)	3.0 (0, 10.0)	0.51
FreKAQ (0-36) (IQR)	2.0 (1.0, 8.5)	4.5 (1.0, 9.0)	0.49
CSI-9 (0-36) (IQR)	3.5 (1.0, 5.0)	6.0 (2.3, 9.0)	0.10
Inappropriate FTA (%)	4 (17.4%)	25 (24.2%)	0.60
β angle (°) (SD)	88.7 (1.6)	89.0 (1.5)	0.42
Knee extension ROM (°) (IQR)	-5.0 (-10.0, 0)	0 (-5.0, 0)	0.06
Knee flexion ROM (°) (SD)	122.5 (10.1)	123.7 (9.8)	0.59
10MWT (s) (SD)	6.2 (1.1)	7.1 (1.6)	0.04*
OKS (0-48) (IQR)	42.5 (39.0, 44.5)	41.0 (38.0, 45.0)	0.26

Data in the table are presented using N (%) for nominal variables, and for continuous variables, the mean ± standard deviation (SD) is used when the data follow a normal distribution, while the median and inter-quartile range (IQR) is reported when the distribution is non-normal. BMI, body mass index; PCS, pain catastrophizing scale; FreKAQ, Fremantle Knee Awareness Questionnaire; CSI-9, The short form of Central Sensitization Inventory; ROM, range of motion; 10MWT, 10-Meter Walk Test; OKS, Oxford Knee Score. Inappropriate FTA was defined as femorotibial angle > 178°or < 170°.

$p < 0.05$*
